# The Effect of Standardized Hospitalist Information Cards on the Patient Experience: a Quasi-Experimental Prospective Cohort Study

**DOI:** 10.1007/s11606-022-07674-3

**Published:** 2022-06-01

**Authors:** Muhammad Hasan Abid, David J. Lucier, Michael K. Hidrue, Benjamin P. Geisler

**Affiliations:** 1 Harvard Medical School, Boston, MA USA; 2 Massachusetts General Hospital/Massachusetts General Physicians Organization, 55 Fruit St, Boston, MA 02114 USA; 3 Institute for Healthcare Improvement, Boston, MA USA; 4 Armed Forces Hospitals Taif Region, Taif, Kingdom of Saudi Arabia; 5 Institute for Medical Information Processing, Biometry, and Epidemiology, Ludwig Maximilian University, Munich, Germany

**Keywords:** patient experience, patient satisfaction [MeSH], physician-patient relations [MeSH], communication [MeSH], patient-reported outcome measures [MeSH], patient-centered care [MeSH]

## Abstract

**Background:**

Communication with clinicians is an important component of a hospitalized patient’s experience.

**Objective:**

To test the impact of standardized hospitalist information cards on the patient experience.

**Design:**

Quasi-experimental study in a U.S. tertiary-care center.

**Participants:**

All-comer medicine inpatients.

**Interventions:**

Standardized hospitalist information cards containing name and information on a hospitalist’s role and availability vs. usual care.

**Main Measures:**

Patients’ rating of the overall communication as excellent (“top-box” score); qualitative feedback summarized via inductive coding.

**Key Results:**

Five hundred sixty-six surveys from 418 patients were collected for analysis. In a multivariate regression model, standardized hospitalist information cards significantly improved the odds of a “top-box” score on overall communication (odds ratio: 2.32; 95% confidence intervals: 1.07–5.06). Other statistically significant covariates were patient age (0.98, 0.97–0.99), hospitalist role (physician vs. advanced practice provider, 0.56; 0.38–0.81), and hospitalist-patient gender combination (female-female vs. male-male, 2.14; 1.35–3.40). Eighty-seven percent of patients found the standardized hospitalist information cards useful, the perceived most useful information being how to contact the hospitalist and knowing their schedule.

**Conclusions:**

Hospitalized patients’ experience of their communication with hospitalists may be improved by using standardized hospitalist information cards. Younger patients cared for by a team with an advanced practice provider, as well as female patients paired with female providers, were more likely to be satisfied with the overall communication. Assessing the impact of information cards should be studied in other settings to confirm generalizability.

**Supplementary Information:**

The online version contains supplementary material available at 10.1007/s11606-022-07674-3.

## INTRODUCTION

Patient centeredness is one of the six domains of healthcare quality as defined by the Institute of Medicine.^1–4^ Good communication between patients and clinicians is an integral part of the patient experience.^3–6^ Ineffective communication has been linked to decreased treatment adherence and increased readmissions, medico-legal risks, increased mortality, and decreased willingness to return and recommend.^7^

It remains unclear if and how standardizing the patient encounter can improve communication.^8^ One approach, business cards^6,9^ or “face cards” that include photos,^10–15^ has been studied in inpatient settings as one possible standardized communication tool to improve patients’ recognition of their clinical team, assist in recalling their names, and improve their knowledge of hospitalists’ roles and responsibilities. Results for improving patient-hospitalist communication, patient satisfaction, trust, and agreement with providers varied based on the setting where they were implemented, leading to questions about their utility.

As part of a quality improvement program, we aimed to implement standardized hospitalist information cards (SHICs) during the initial patient interaction, with the goal to improve the patients’ perception of the overall communication.

## METHODS

### Overview

A quasi-experimental survey design was used to evaluate the impact of SHICs on patients’ rating of hospitalists’ communication skills. An inpatient communication experience survey was used as the primary instrument to collect patients’ perceptions of the quality of the communication with their hospitalist(s) across three hospital medicine floors. Two floors were selected for the intervention and a third floor served as a control. This project was undertaken as a quality improvement initiative at Massachusetts General Hospital. At our institution, quality improvement projects are exempted from a full review by the institutional review board per their policies; this determination was made by the Department of Medicine’s Chair of Quality and Safety (D.J.L.) using a standardized checklist.

### Setting

The study was conducted in a 1,011-bed tertiary-care center in Boston, Massachusetts. The Hospital Medicine Unit (HMU) consists of approximately 145 attending physicians (MD/DOs) and advanced practice providers (APPs)—nurse practitioners (NPs) and physician assistants (PAs)—collectively called hospitalists, who provide clinical care to an estimated average of 175 inpatients daily across twelve different floors, as well as to emergency room patients already admitted but still waiting for a free bed. Three of these floors are organized so that individual hospitalists will have the majority of patients under their care on that unit. Two of these “regionalized” floors were chosen to test the intervention. MD/DOs on these floors cared for approximately nine patients per day when working by themselves, and up to fourteen patients per day when working with APPs. APPs cared for approximately six patients per day with a supervising MD. All clinicians on average spend 4 to 5 days on service, followed by a variable number of days off service. In our HMU, there is generally a high proportion of patients who request or who are offered phone calls to relatives. However, the proportion of white boards in the patients’ rooms being used might vary more between MGH hospitalists: in some cases, nurses write team names on the board; in other cases, hospitalists use the white board to remind patients and others of their name and role and how to contact them.

### Participants/Study Population

All patients admitted to the dedicated HMU floors under an HMU attending were eligible to receive a survey about the communication. Exclusion criteria for surveying consisted of patients (1) who were non-English-speaking; (2) with an altered mental status or encephalopathy, including from delirium or dementia; (3) with expressive or receptive speech aphasia; (4) withdrawing from substances or with behavioral dysregulation; (5) receiving end-of-life care or those who were critically ill; (6) who were asleep or did not want to be disturbed; (7) not physically in their room at the time of surveying; and (8) who explicitly declined to participate.

### Intervention

We created two-sided, 5.5″ × 4.25″ SHICs for MD/DOs and APPs, which included space to write the hospitalist’s name, a description of their role, their schedule, and how to contact the hospitalist or the team. We incorporated feedback from various stakeholder groups in an iterative fashion to create a final version of SHICs (see Appendix). Hospitalists were instructed to hand out SHICs to all of their patients when working on the two intervention floors, and to not hand them out when working on any other floor. SHICs were placed in a visible location in the floor work rooms to facilitate easy use. Hospitalists were intentionally not trained on how to use the SHIC; they were only instructed to utilize the SHICs as a basis for discussing their own role and responsibilities, and to use one with every new patient they met on one of the two intervention floors.

### Data Collection

We used the inpatient communication experience survey once a week between November 2018 and March 2019 on the three floors, one non-intervention floor and two intervention floors. However, on the two latter floors, the SHICs were only implemented from January 1, 2019, onwards—dividing the study duration into a pre-intervention (November and December 2019) and a post-intervention phase (January 1 to March 31, 2019). Demographic data collected included the patient’s age and gender as well as the hospitalist(s’) role(s) and gender(s). We further excluded responses from patients on the intervention floor who did not receive an SHIC, responses for hospitalists who had one or more survey responses in only the pre-intervention or post-intervention period, and responses where one of the co-authors was in the hospitalist role. We did not collect data on other means to convey information to patients, family, or caregivers such as white board use phone calls.

### Survey Design and Endpoints

We used CI-CARE patient questionnaires^9^ to create the inpatient communication experience survey (see Appendix). We asked patients about different aspects of communication, asked them to rank the overall communication of their hospitalist, and asked them the usefulness of the SHICs (if they received one). If they had multiple hospitalists, they were primed to think about the one they were working with currently. For patients cared for by both an APP and an MD/DO, two separate surveys were conducted. The primary outcome was the overall communication score, measured via a five-point Likert scale (“Excellent,” “Very Good,” “Good,” “Fair,” and “Poor”). We converted the responses into a binary score of “top box” (“excellent” vs. all other options) in line with the bulk of analyses of patient satisfaction and experience. For patients that received a card, a secondary outcome was their perception of the overall utility of the card based on a three-point scale—“Yes, it was helpful,” “It made no difference,” and “No, it was not helpful.”

The qualitative free-text comments of the patients who found SHICs useful were analyzed using inductive coding to create a taxonomy of themes and subcategories. Two authors (D.J.L. and B.P.G.) used the taxonomy to independently score the free-text comments. Discrepancies in scoring were adjudicated by a third author (M.H.A.) to arrive at a final score for each comment.

### Statistical Analysis

The primary outcome was prespecified as a dichotomized “top-box” vs. not “top-box” score. Descriptive statistics were used to summarize the data and the chi-square test was used to compare top-box rates across sample characteristics. Multivariate logistic regression analysis was performed to control for effect-modifying factors and estimate the effect of SHICs on patients’ perceptions of hospitalists’ communication skills. The regression model controlled for patient age, patient-hospitalist gender combinations, hospitalist type (MD/DO vs. APP), study period (before or after the intervention), study group (intervention vs. control), and the interaction of the two. The estimate for the interaction term represents the effect of SHICs. Due to responses from patients treated by the same hospitalist being more likely to be similar than responses from patients treated by different hospitalists, we specified a generalized estimating equation to account for the clustering of patient response under a clinician. APP survey responses were considered independent from the corresponding MD/DO response. All statistical tests were two-sided, and the alpha level was set at 0.05. The base case analysis were performed using SAS 9.4 (SAS Institute Inc., Cary, NC, USA).

## RESULTS

**Figure 1:**
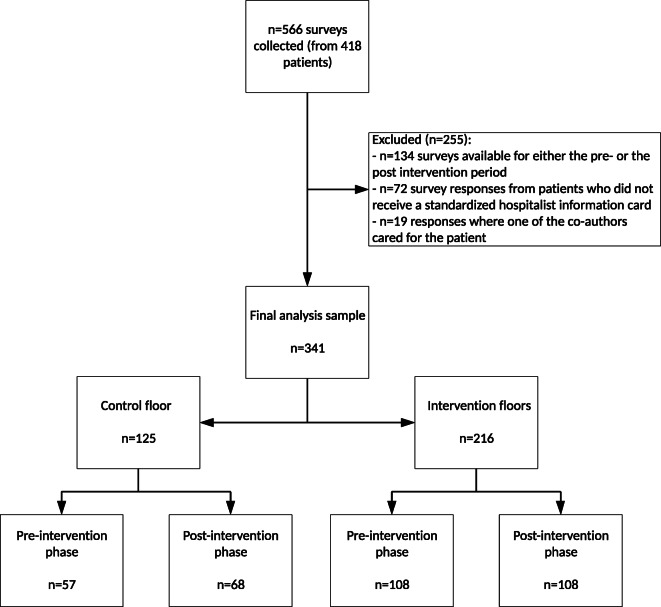
**Flow chart of the number of participants approached, included, and excluded.**

A total of 566 inpatient communication experience surveys were collected from 418 unique patients during the measurement period. There were 148 “shared” visits where both an APP and an MD/DO saw the patient (26%). After excluding patients who were not given a SHIC, patients cared for by one of the co-authors, and hospitalists who were not present in both the pre-intervention and post-intervention time periods, 341 surveys remained. See Figure 1 for a breakdown of the reasons for exclusion. The mean patient age was 62.4 (standard deviation: 18.8) years and 52% were female. Of note, our sample only included one male APP. Of the 341 patients, 216 (63%) were on the intervention floors and 125 (37%) were on the control floor. In univariate analyses, we found statistically significant difference in overall communication score by hospitalist gender (*p* = 0.003), patient-hospitalist gender combination (*p* = 0.001), and hospitalist type (*p* = 0.002), but no statistically significant difference by the three floors (*p* = 0.124). On the intervention floors, the overall communication score increased from 52.8% in the pre-intervention phase to 70.4% in the post-intervention phase, a difference of 17.6% (*p* = 0.008). The overall communication score for the non-intervention floor was 49.1% in the pre-intervention phase and 54.4% in the post-intervention phase, with a statistically non-significant difference (*p* = 0.556). See Table 1.

**Table 1:** Baseline Characteristics and Association with the “Top Box” Communication Score

**Covariate**	**Distribution of patients,** ***n*** **(%)**	“Top-box” score on communication*	
		***n*** **(%)**	***p*** **value**
Patient age, mean (SD)	62.4 (18.8)		
Patient gender			0.135
Male	165 (48.4)	89 (53.9)	
Female	176 (51.6)	109 (61.9)	
Hospitalist gender			0.003
Male	156 (45.8)	74 (47.4)	
Female	185 (54.2)	124 (67.0)	
Hospitalist-patient gender			0.001
Male hospitalist-male patient	75 (22.0)	34 (45.3)	
Male hospitalist-female patient	81 (23.7)	40 (49.4)	
Female hospitalist-male patient	90 (26.4)	55 (61.1)	
Female hospitalist-female patient	95 (27.9)	69 (72.6)	
Hospitalist type			0.002
MD/DO	196 (57.5)	100 (51.0)	
APP	145 (42.5)	98 (67.6)	
Floor			0.124
Floor 1	145 (45.5)	93 (64.1)	
Floor 2	71 (20.8)	40 (56.3)	
Floor 3	125 (36.7)	65 (52.0)	
Intervention floors			0.008
Pre-intervention period	108 (50.0)	57 (52.8)	
Post-intervention period	108 (50.0)	76 (70.4)	
Control floors			0.556
Pre-intervention period	57 (45.6)	28 (49.1)	
Post-intervention period	68 (54.4)	37 (54.4)	

*SD* standard deviation, *MD/DO* medical doctor/doctor of osteopathic medicine, *APP* advanced practice provider (nurse practitioner or physician assistant)

*n (%) refers to the number of patients (% of patients) with a "top box" score on a specific category. For example, for the male row in the patient gender category, 89 (53.9) indicates there were 89 patients who chose a "top box" score, and these represent 53.9% of the male respondents (165 in this case). The *p* values are based on univariate analyses with a chi-square test and test if the difference in the “top-box” scores for a covariate is statistically significant

Table 2 presents the results of the multivariate regression. We found that higher patient age was associated with a lower communication score (odds ratio [OR] = 0.98, 95% confidence interval [CI]: 0.97; 0.99). Solo hospitalists received a lower communication score compared to teams that included APPs (OR = 0.56; 95% CI: 0.38; 0.81). Female patients rated female hospitalists better than they did male hospitalists (OR = 2.14, 95% CI: 1.35; 3.40). The intervention group had a statistically higher communication score than the control group in the baseline period (OR = 1.95, 95% CI: 1.01; 3.74). The communication score for the control group did not change significantly between the baseline and the follow-up periods (OR = 0.97, 95% CI: 0.53; 1.78). The interaction term, which measures the impact of the intervention beyond difference in baseline tendencies and temporal changes, was statistically significant (OR = 2.32, 95% CI: 1.07; 5.06).

**Table 2:** Estimating the Association of Standardized Hospitalist Information Card and Patient’s “Top-Box” Overall Communication Score

**Covariate**	**Odds ratio**	
	**Estimate**	**(95% CI)**
Patient age (in years)	0.98	(0.97–0.99)
Hospitalist type (reference: APP)	0.56	(0.38–0.81)
Hospitalist and patient gender (reference: male hospitalist and female patient)		
Male hospitalist-male patient	0.83	(0.44–1.56)
Female hospitalist-male patient	1.07	(0.70–1.64)
Female hospitalist-female patient	2.14	(1.35–3.40)
Post-intervention period (reference pre-intervention)	0.97	(0.53–1.78)
Intervention group (reference: control group)	1.95	(1.01–3.74)
Post-intervention period * intervention group (interaction term)	2.32	(1.07–5.06)

*95% CI* 95% confidence interval, *APP* advanced practice provider (nurse practitioner or physician assistant)

Among patients who received SHICs (*n* = 141), 87% (*n* = 122) found them to be useful, and 13% (*n* = 22) mentioned that it made no difference. The inductive coding analysis of these *n* = 122 patients demonstrated that perceived usefulness was most related to knowing how to contact the hospitalist (39.3%), knowing the schedule of the hospitalist (25.4%), feeling like communication was enhanced (21.3%), helping to identify the hospitalist (20.5%), and aiding in understanding the care process (20%). See Appendix.

## DISCUSSION

Patients’ perception of the overall communication improved in this study with standardized hospitalist information cards. Most patients who received a card found them useful, particularly on how to contact their hospitalist. In addition, teams that included APPs were found to be more highly rated on overall communication than solo hospitalists. The combination of patient-clinician gender was, unexpectedly and independent of SIHC, found to have significant effects on perceptions of overall communication, with female patients rating female hospitalists highest compared to male patients rating male hospitalists. To our knowledge, this is the first study to show improvement in the hospitalist-patient communication through exposure to SHICs and to identify the patient-hospitalist genders as important independent predictors of perception about communication.

The primary goal of effective patient-hospitalist communication is to convey appropriate information about their general care process and the specific care plan in a respectful and courteous manner, as well as answering patients’, relatives’, and other caregivers’ questions. Setting expectations around the general care process, for example the hospitalists’ schedule or how to contact them, can help to build trust in the patient-hospitalist relationship and might ultimately improve the patient experience. From a quality improvement and patient safety standpoint, an excellent hospitalist-patient communication could potentially add value to the coproduced healthcare service^2^ provided to the patient and adds a safety net to prevent medical errors and, possibly, legal exposure. SHICs can serve as a physical aid that could help improve and standardize the initial hospitalist-patient interaction through scripted information and setting expectations around the care process.

There was a strong patient-hospitalist gender-gender interaction with a highly significant likelihood of female patients rating female hospitalists as excellent at overall communication compared to all other combinations. The female hospitalist-male patient and male hospitalist-female patient gender permutations were also better than the male hospitalist-male patient gender combination on the overall communication skill “top-box” score, but were not statistically significant, suggesting the female-female gender pairing results in better perception of overall communication. This is surprising and contrary to what Apker et al. have demonstrated on the hospitalist-patient gender interaction and its effect on communication quality, where male hospitalists were significantly rated higher than the female hospitalists.^16^ However, in our sample, there was only one male APP, and because of the small sample size, we could not test an interaction of APP and gender. Our results suggest further research is needed in this area to better understand how gender impacts communication with patients.

We also identified an independent effect of the presence of an APP on patients’ rating of the communication. One possible explanation for this finding is the lower average daily patient census being cared for by the hospitalist APPs compared to hospitalist MD/DOs over the same time period. A smaller census may result in hospitalist APPs spending some more time in their patient encounters than hospitalist MD/DOs are able to spend. Future research would be helpful to further elucidate the impact of census on communication independent of role group. Patients cared for in shared visits, i.e. seen by both an APP and an MD/DO, receive at a minimum two daily visits, one from the supervising MD/DO and an often longer one from the APP; this might possibly make patients rate the communication with the APP better than the one with a physician. APPs might also receive more formal or practical training in communication that MD/DOs do not, which could explain some of the results. Finally, the gender interaction might play a role, as our sample only contained one male APP.

While the card itself was standardized, we did not standardize how they should be used, i.e., how hospitalists should describe the cards and the role of the information contained on them. This has the advantage of making this intervention relatively simple, inexpensive, and easy to implement. However, it is conceivable to develop a script; i.e., standardizing how the card should be described to the patient could further improve their effectiveness or at least efficiency.

Our study had several strengths, including the quasi-experimental study design in a real-world setting, the relatively high number of surveys collected in a single-institution setting, and the combination of qualitative and quantitative methods. Adding to the existing body of literature,^6,9–15^ our study is the first to study both APPs and MD/DOs as well as the patient-hospitalist gender combinations. Only one prior study found different effects of information or face cards between resident physicians of a different gender.^9^ On a broader scope, there are trade-offs between the sometimes more selected and therefore possibly somewhat artificial composition of randomized controlled trials and real-world evidence. Nevertheless, real-world studies such as the present one should be conducted and evaluated as rigorously as possible.

This quality improvement study is subject to several limitations. First, there may be a chance for residual patient- or hospitalist-level confounders due to the quasi-experimental design, such as the frequency of phone calls to family members or the use of white boards. However, patient assignments to floors were arbitrarily chosen by the hospital’s non-clinical admitting department staff and were not influenced by this initiative, and we also controlled for some important covariates in our adjusted analyses. Second, we neither collected data on patients who declined to participate nor assessed the extent to which the intervention was implemented in the experimental and the control group, which might have led to potential contamination and, consequently, an underestimation of the true difference. However, the nature of the intervention is to only study SHICs as a suggested tool to improve communication. Third, the initiative was conducted in a single center with a limited sample size over a short period and no a priori sample size calculation, and thus, they are not generalizable. However, our findings can and should be attempted to be replicated in other institutions to test their generalizability, and our data can be used to calculate appropriate sample sizes. Fourth, a substantial part of our respondents was either not available during either the pre- or the post-intervention period or did not receive a standardized hospitalist information card, which might have led to selection bias. However, it may be helpful for future studies to anticipate the effect of this on the sample size. Fifth, important factors that contribute to unconscious bias and will affect perceptions of communication, such as patient and hospitalist race, ethnicity, primary language, perceived socioeconomic status, and perceived hospitalist age, were not collected. However, these factors might affect communication, so not adjusting for them leaves us with the overall effect. Finally, there was only one male APP in our sample. However, given this is a single-institution study, the generalizability needs to be confirmed regardless.

In summary, patient-hospitalist communication may be improved by using a simple and inexpensive intervention, providing the patient with a standardized hospitalist information card during the initial hospitalist-patient interaction. Team-based care with advance practice providers might also be associated with a higher satisfaction of patients with the overall communication. Female patients may have a preference for how they communicate with female hospitalists. However, further studies are required in other settings to assess for the implementation of the standardized hospitalist information card and improvement in the quality of hospitalist-patient communication.

## Supplementary Information

ESM 1 (DOCX 325 kb)
