# Supplementary material for: The Effect of Standardized Hospitalist Information Cards on the Patient Experience: a Quasi-Experimental Prospective Cohort Study
Source: J Gen Intern Med. 2022 Jun 1;37(15):3931–6. doi: 10.1007/s11606-022-07674-3 (PMC9640479; doi:10.1007/s11606-022-07674-3)
Supplement: Supplementary file 1 — (DOCX 325 kb) [file 11606_2022_7674_MOESM1_ESM.docx]

Appendix

The Effect of Standardized Hospitalist Information Cards on the Patient Experience

A Quasi-Experimental Prospective Cohort Study

Muhammad Hasan Abid, MBBS, MHQS_­_^1-4^; David J. Lucier, MD, MBA, MPH^1,2^; Michael K. Hidrue, PhD^2^; Benjamin P. Geisler, MD, MPH^1,2,5^

^1^Harvard Medical School, Boston, Massachusetts; ^2^Massachusetts General Hospital/Massachusetts General Physicians Organization, Boston, Massachusetts; ^3^Institute for Healthcare Improvement, Boston, Massachusetts; ^4^Armed Forces Hospitals Taif Region, Taif, Kingdom of Saudi Arabia; ^5^Institute for Medical Information Processing, Biometry, and Epidemiology, Ludwig Maximilian University, Munich, Germany.

Inpatient Communication Experience Survey

Figure 1: Standardized Hospitalist Information Card


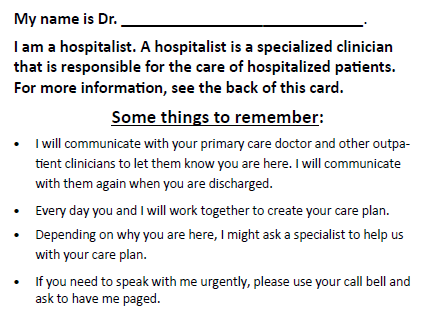

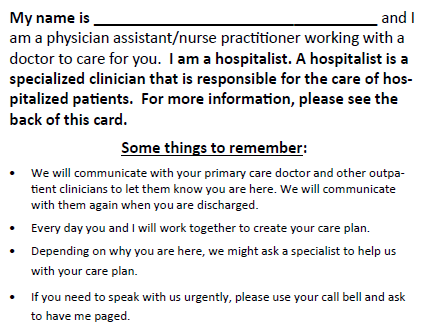

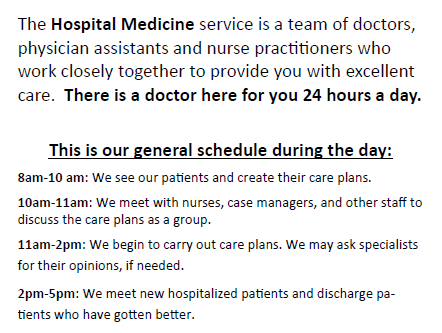


MD Front

NP/PA Front

Both Back

Table 1: Marginal Effect for the Difference in Difference. This table lists the difference-in-difference (“marginal effect”) with lower and upper confidence interval (CI) and p-value.

|  | Margin | Lower CI | Upper CI | p-value |
| --- | --- | --- | --- | --- |
| Baseline Prob_Treatment Group | 0.5307352 | 0.4332392 | 0.6282313 | 0 |
| Follow-up Prob_Control Group | 0.7035621 | 0.6146284 | 0.7924959 | 0 |
| Change in Prob for treatment Group | 0.1728269 | 0.0443883 | 0.3012655 | 0.088 |
| Baseline Prob_Control Group | 0.4911662 | 0.35926 | 0.6230724 | 0 |
| Follow-up Prob_Treatment Group | 0.5424417 | 0.4214358 | 0.6634477 | 0 |
| Change in Prob for Control Group | 0.0512756 | -0.1251528 | 0.2277039 | 0.57 |
| Impact of Intervention (diff-in-diff) | 0.1215513 | -0.0974115 | 0.3405142 | 0.277 |

Table 2: Inductive Coding Analysis. Reasons for Why Patients Found Standardized Hospitalist Information Cards Useful.

| **Domain** | **Key Word** | **Proportion Mentioned** |
| --- | --- | --- |
| Emotions | Comfortable | 13% |
| Care Process | Well-informed | 15% |
|  | Aids Understanding | 20% |
| Logistics/Communication | Role | 10% |
|  | Communication | 21% |
|  | Identify | 21% |
|  | Contact | 39% |
|  | Scheedule | 25% |
